# Serious kidney disease in pregnancy: an Australian national cohort study protocol

**DOI:** 10.1186/s12882-019-1393-z

**Published:** 2019-06-25

**Authors:** Nadom Safi, Elizabeth Sullivan, Zhuoyang Li, Mark Brown, William Hague, Stephen McDonald, Michael J. Peek, Angela Makris, Angela M. O’Brien, Shilpanjali Jesudason

**Affiliations:** 1 Faculty of Health, University of Technology Sydney, 235 Jones Street Ultimo, Sydney, NSW 2007 Australia; 2 Faculty of Health and Medicine, University of Newcastle, 130 University Drive, Callaghan, 2308 NSW Australia; 3 Department Renal Medicine and Medicine, St. George Hospital and University of New South Wales, Kogarah, Sydney, Australia; 4 Robinson Research Institute, University of Adelaide, Women’s and Children’s Hospital, Adelaide, 5006 SA Australia; 5 ANZDATA Registry, South Australia Health and Medical Research Institute, Adelaide, Australia; 6 Adelaide Medical School, University of Adelaide, Adelaide, SA Australia; 7 Central and Northern Adelaide Renal and Transplantation Service (CNARTS), Royal Adelaide Hospital, Adelaide, SA Australia; 8 The Canberra Hospital, The Australian National University, Bdg 11, Level 2, Yamba Dve, Garran, Canberra, 2605 ACT Australia; 9 University of Western Sydney and the University of New South Wales, Sydney, Australia

**Keywords:** Cohort study, Population, Pregnancy, Pregnancy outcome, Chronic kidney disease, Acute kidney injury, Kidney transplantation, Renal dialysis

## Abstract

**Background:**

Maternal kidney disease (acute kidney injury (AKI), advanced chronic kidney disease (CKD), dependence on dialysis or a kidney transplant) has a substantial impact on pregnancy, with risks of significant perinatal morbidity. These pregnancies require integrated multidisciplinary care to manage a complex and often challenging clinical situation. The ability to deliver optimal care is currently hindered by a lack of understanding around prevalence, management and outcomes in Australia. This study aims to expand an evidence base to improve clinical care of women with serious kidney impairment in pregnancy.

**Methods/design:**

The “Kidney Disease in Pregnancy Study” is a national prospective cohort study of women with stage 3b-5 CKD (including dialysis and transplant) and severe AKI in pregnancy, using the Australasian Maternity Outcomes Surveillance System (AMOSS). AMOSS incorporates Australian maternity units with > 50 births/year (*n* = 260), capturing approximately 96% of Australian births. We will identify women meeting the inclusion criteria who give birth in Australia between 1st August 2017 and 31st July 2018. Case identification will occur via monthly review of all births in Australian AMOSS sites and prospective notification to AMOSS via renal or obstetric clinics. AMOSS data collectors will capture key clinical data via a web-based data collection tool. The data collected will focus on the prevalence, medical and obstetric clinical care, and maternal and fetal outcomes of these high-risk pregnancies.

**Discussion:**

This study will increase awareness of the issue of serious renal impairment in pregnancy through engagement of 260 maternity units and obstetric and renal healthcare providers across the country. The study results will provide an evidence base for pre-pregnancy counselling and development of models of optimal clinical care, clinical guideline and policy development in Australia. Understanding current practices, gaps in care and areas for intervention will improve the care of women with serious renal impairment, women with high-risk pregnancies, their babies and their families.

**Electronic supplementary material:**

The online version of this article (10.1186/s12882-019-1393-z) contains supplementary material, which is available to authorized users.

## Background

Chronic Kidney Disease (CKD) is an under-recognised, serious and increasing major health problem, which disproportionately affects Indigenous peoples and lower socioeconomic groups [1–3] . The prevalence of CKD amongst Australian mothers is rising due to the population rise in diabetes, obesity and older age in pregnancy [4]. Over 5% of Australian women of child-bearing age have albuminuria or abnormal estimated GFR (eGFR) indicating CKD [2]. In Australia in 2016, 25% of all women with a functioning renal transplant and 51% of all female incident end-stage kidney disease (ESKD) cases were in women of reproductive age (15–44 years) [5]. Parenthood is a major concern for women with CKD, and women experience important fears and uncertainties about risks to their own and their baby’s health [6, 7]. Women of childbearing age with CKD may face difficult decisions about parenthood while experiencing significant comorbidity and impairment to activities of daily living. Navigating paths to parenthood via complex shared decision-making can be challenging and accurate outcome data would enable evidence-based risk stratification [7].

There is a knowledge gap regarding the burden of CKD in pregnancy in the Australian context. Determining the prevalence of kidney disease in pregnancy has not been easy, due to varying definitions, changing staging systems and poor capture of renal markers (serum creatinine and urine protein) in pregnancy cohorts [8, 9]. The Australia and New Zealand dialysis and transplant registry (ANZDATA) reports pregnancy rates for chronic dialysis and transplant recipients of 3.3/1000 person-years and 20/1000 person-years respectively but is likely under-reported [10–12]. Rates of advanced stage CKD (Stage 3b-4), and non-chronic Acute Kidney Injury (AKI) occurring in pregnancy in Australia are unknown. This information is important when developing maternity and renal services.

There is currently no data on how significant kidney disease in pregnancy (CKD stage 3b-5, dialysis, transplant or AKI) impacts upon pregnancy care and how it is managed in Australia. Registry and perinatal datasets currently active in Australia collect some data items and past analyses of the ANZDATA registry have provided some information on pregnancy rates and basic pregnancy outcomes [10, 13]. However, these provide minimal insights into obstetric and perinatal adverse events, therapy and interventions required during pregnancy, models of care delivered, and utilisation of health care resources including hospitalisation and other events.

This knowledge is important to obtain as pregnancy in women with CKD is associated with worse obstetric and perinatal outcomes including preterm birth, small for gestational age, and pre-eclampsia and gestational hypertension [14, 15]. The risk is increased, even in women with Stage 1 CKD [16, 17] and continues to rise as CKD stage advances [15, 18, 19]. Pre-conception eGFR< 40 ml/min, poorly controlled hypertension and proteinuria are factors associated with higher rates of obstetric and perinatal complications [20–23]. As pre-conception renal function worsens, the risk of “stage shift” or deterioration in renal function also increases, and may necessitate dialysis during pregnancy [9, 24, 25].

Pregnancies in women receiving chronic dialysis are rare due to the reduced fertility of women with advanced kidney failure, and are particularly challenging in terms of maternal care [8]. The recognition that intensive dialysis improves outcomes has led to changes in clinical practice and counselling for women with advanced CKD [19, 24, 26, 27]. Australian women receiving chronic dialysis have a 27% fetal loss rate compared with 7% among all pregnancies [10]. Among live births, 75% are preterm and more than 50% of such babies have birth weights less than the 10th centile [10]. Outcomes are better for women who commence dialysis after conception compared with those who conceive while receiving chronic dialysis [10, 28]. Even after successful kidney transplantation, these pregnancies continue to have high rates of pre-eclampsia (> 25%), prematurity and poor fetal growth (> 50%) [12, 13, 29]. Worse graft function (serum creatinine > 110 μmol/L) during pregnancy can predict risk of pre-eclampsia and kidney function trajectory including significantly higher rates of graft loss within 3 years of giving birth [29].

The incidence of Acute Kidney Injury (AKI) in pregnancy has decreased in recent years in developed countries, mainly due to improvement in peripartum sepsis care [30]. However, AKI still represents a potentially catastrophic event in pregnancy, caused predominantly by pre-eclampsia [31]. There is also enhanced awareness and understanding of newer conditions such as atypical haemolytic uraemic syndrome [32]. AKI in one pregnancy is associated with worse outcomes in the next [33]. Approximately 1/10,000 pregnancies are complicated by AKI that requires dialysis, which was associated with poor perinatal outcomes and increased maternal death rate in one Canadian study [34]. There are no Australian data regarding frequency, causes and outcomes of pregnancy-related AKI, and this remains an important area to investigate.

Severe CKD and AKI in pregnancy, while uncommon, cause a high burden of maternal and perinatal morbidity [14, 15, 34]. A lack of information regarding outcomes for women with these conditions in Australia including the maternal mortality and morbidity, model of care, perinatal outcomes, and access to services, presently limits the health service providers’ ability to counsel and manage affected women. The model of care required for such high risk pregnancies may not be equitably available to all women at higher risk, particularly those who are Indigenous, geographically isolated, from a lower socioeconomic status (SES) or from a culturally and linguistically diverse (CALD) group.

The aim of this study is to determine the Australian prevalence, pregnancy-related and pre-existing maternal and renal morbidity and perinatal outcomes associated with serious maternal kidney disease in pregnancy; and to describe patterns of presentation, pregnancy management and models of care and adequacy of healthcare delivery in these pregnancies. We will achieve this by development of a new framework for capture of maternal and fetal outcomes for cases of significant kidney disease in pregnancy, using the existing Australasian Maternity Outcomes Surveillance System (AMOSS) research platform which is embedded in many maternity units across Australia. We hypothesise that mothers with CKD and AKI will have a substantial pregnancy related and pre-existing medical morbidity, and that babies born to these mothers will have higher prematurity and consequent perinatal morbidity. Adverse outcomes may be related to modifiable factors in pre-pregnancy and antenatal care, including access to specialised services, improved blood pressure management and dialysis treatment, fetal surveillance and timing of delivery. The study findings will inform a guideline and policy development and improve counselling for this high risk cohort.

## Methods/design

### Study objective

Using the existing AMOSS maternity surveillance system embedded in 260 maternity hospitals in Australia we will identify cases of significant CKD (Stage 3b-5 including dialysis and transplant) and severe AKI in pregnancy.

1. To determine the prevalence, and geographical distribution.
2. Utilise survey data to determine pregnancy outcomes (maternal and perinatal), renal outcomes, models of care, and maternity management

### Study design

A national, prospective cohort study using the Australasian Maternity Outcomes Surveillance System (AMOSS). The AMOSS methods have been described in detail elsewhere [35, 36]. In brief, AMOSS is an established surveillance and research system across maternity units in Australia and New Zealand to study rare but clinically important and severe conditions in pregnancy, which place a disproportionate burden on women, their families and healthcare systems. AMOSS has access to almost all (95% [260/275]) eligible Australian maternity units with more than 50 births/year, capturing an estimated 96% of women giving birth in a hospital in Australia.

### Study population, case definition and inclusion criteria

The study population will include all women giving birth in participating Australian AMOSS sites between 1st August 2017 and 31st July 2018. Cases of serious kidney disease giving birth in participating Australian AMOSS sites during the study period will be collected. Table 1 shows the inclusion criteria. Birth is defined as the birth of one or more live or stillborn infants of at least 20 weeks gestation or at least 400 g birthweight. Women not giving birth (e.g. pregnancy ending in miscarriage or termination of pregnancy < 20 weeks gestation) or with early-stage CKD and less severe AKI will be excluded from the study.

**Table 1:** Inclusion criteria

All women in Australia who gave birth:	
a. Between 1 August 2017 and 31 July 2018; AND b. At least 20 weeks’ gestation and/or at least 400 g birth weight; AND c. Who present with at least one of the following criteria:	
1. A working kidney transplant (all women regardless of kidney transplant function)	
2. Receiving any long-term dialysis before conception and continuing any dialysis during pregnancy.	
3. Starting any dialysis during pregnancy (any dialysis - either once off, temporary or permanent dialysis)	
4. Known pre-conception eGFR < 45 ml/min/1.73m^2^ (known to have a serum creatinine > 130–150 μmol/L before conception, regardless of the serum creatinine reading during pregnancy)	
5. Newly identified renal function impairment with any serum creatinine reading of > 150 μmol/L on 2 readings at least 24 h apart during pregnancy	

### Assessment of renal function in pregnancy – selection of inclusion criteria

Serum creatinine is a widely used marker of renal function in non-pregnant patients, which varies depending on muscle mass, age, and ethnicity. Renal function in non-pregnant patients is evaluated by calculating the estimated glomerular filtration rate (eGRF), with formulae derived from populations using gender, age, ethnicity and serum creatinine measurement. The eGFR is most commonly calculated using the CKD-EPI formula and is routinely provided when reporting serum creatinine in the adult population by laboratories in Australia [37]. However, eGFR formulae are not valid for use during pregnancy, where serum creatinine usually falls due to renal physiological changes, and where a serum creatinine > 80-90 mmol/L usually reflects a degree of renal impairment. Therefore, in this study, eGFR will only be used for pre-pregnancy serum creatinine values. As shown in Table 2, in women aged 18–45 years, a pre-pregnancy serum creatinine of 130–150 micromol/L or more will reflect eGFR < 45 ml/min/1.73m^2^ (CKD Stage 3b or higher) [38]. The data collector will obtain pre-pregnancy serum creatinine measurements wherever available, for all cases.

**Table 2:** Pre-pregnancy serum creatinine and eGFR

Age	eGFR^a^ based on serum creatinine 150 micromol/L
18	44^b^
25	41^b^
30	40^b^
35	39^b^
40	37^b^
45	36^b^
50	35^b^

^a^eGFR(ml/min/1.73m^2^) calculated for adult females at various ages within the childbearing age range, using the CKD-EPI formula [45] without ethnicity coefficients

^b^eGFR < 45 ml/min/1.73m^2^ indicates Stage 3b CKD. Therefore, all women with pre-pregnancy serum creatinine ≥150micromol/L will have CKD Stage 3b or higher

**Fig. 1:**
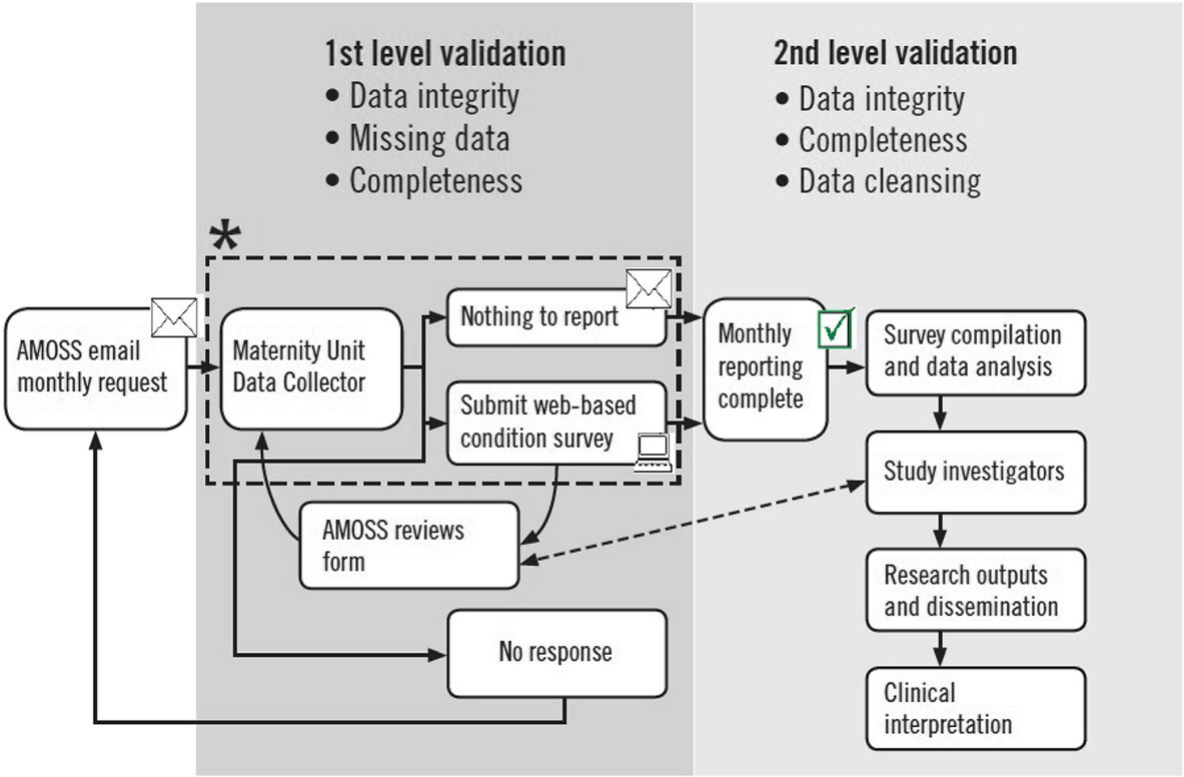
AMOSS surveillance and data collection workflow. *Hospital case identification. Clinical management/Hospital based obstetric and other information systems. Morbidity and/or clinical review meetings. Direct and indirect notification

AMOSS data collectors based in each AMOSS participating site will complete two web-based data collection forms for all women who fulfil the inclusion criteria for the study (Fig. 1). The first survey form is the general AMOSS data collection form utilised in all AMOSS studies, obtaining demographic characteristics such as maternal age, hospital sector, comorbidity, smoking, and BMI, and captured data on medical and obstetric history, obstetric interventions and information about type of care, and location of care. In addition this general survey collects information regarding baby outcomes including birth status, gestation, gender, birthweight, admission to intensive or specialised care units, congenital abnormalities, and neonatal morbidity and mortality (Additional file 1).

The second survey has been developed for this study will capture specific renal information (Additional file 2):

1. General renal history: presentation; the underlying cause of kidney disease; primary diagnosis including kidney biopsy; biochemical data; proteinuria; blood pressure; details of kidney disease management; timing and model of antenatal care; and access to specialist obstetric, obstetric medicine and nephrology services.
2. Transplant history: transplant source; transplant biopsy; anti-rejection treatment and therapeutic monitoring.
3. Dialysis: commencement of dialysis during pregnancy; dialysis modality; vascular access and complications.
4. Medication and other therapies: medication used prior to pregnancy; details of antihypertensive and immunosuppressant medications.
5. Antenatal, birth and postpartum care: blood pressure measurements; antenatal screening and fetal growth scans; antenatal events, including diagnoses of pre-eclampsia, placental abruption; blood transfusion; and birth and post-partum management.

### Study outcomes

Primary outcomes: Prevalence rates of maternal AKI and CKD in the general AMOSS birth (live or stillbirths) cohort as per inclusion criteria. The rate will be expressed as per 1000 maternities based on birth rates for AMOSS hospitals during the study period.

Secondary outcomes will include: 1) Maternal obstetric outcomes and complications – a range of outcomes during antenatal, delivery and post-partum stages; 2) Maternal renal outcomes including renal function, graft function and commencement of dialysis; 3) Adequacy and complications of dialysis therapy; 4) Transplant-related complications including graft dysfunction and infection; 5) Perinatal outcomes including birth outcomes and adverse outcomes especially related to preterm birth (Additional file 3).

### Data collection

Electronic survey data will be collected via an encrypted web-based data management system (the ‘AMOSS system’). Record data will be kept on a secure server with daily backup to an offsite facility. Where a data collector is not able to complete a survey using the web-based survey system, they may handwrite survey responses using the PDF versions of surveys, and scan/send to AMOSS where the survey will be entered on their behalf by the AMOSS team.

AMOSS provides continuous education and support for data collectors before the start of data collection. This is achieved by disseminating the renal study protocol using the AMOSS newsletter, emails, phone calls and presentations at hospitals and conferences. The AMOSS team and investigators will provide support for all data collectors regarding enquiries during the study via emails, phone calls or site visits as requested.

### Participant identification

We will identify study participants through multiple sources: AMOSS surveillance; notification from nephrologists, obstetric physicians and obstetricians; audit committees; and review of routine data collection within hospitals.

### Sample size

Based on current international literature, which estimates CKD Stage 3b-5 rate of 2 to 12 per 10,000; severe AKI requiring dialysis as 1 per 10,000 pregnancies; estimated 295,530 births ≥20 weeks gestation/year in participating Australian AMOSS sites [34, 39–41]; and allowing for potential early pregnancy losses; we estimate identifying between 89 and 384 cases in 1 year.

### Statistical analysis

We anticipate our analysis will largely utilise descriptive statistics (frequency (percentage) for categorical data and mean (standard deviation) or median (interquartile range) for continuous data) will be used to summarise the demographic characteristics, kidney disease data, maternal and baby outcomes of women with advanced CKD, dialysis, transplant and AKI. Cases will be reviewed in depth to map patient care models. Maternal and baby outcomes will be compared with Australian norms. If sample size allows, odds ratios will be estimated via logistic regression for outcomes and adjusted for relevant parameters including demographic data (maternal age, ethnicity); renal function, proteinuria and blood pressure at conception; time from commencement of dialysis or transplant; and dialysis modality and hours (Additional file 4). Variables associated with outcomes in the univariable analysis (*p* < 0.2) and other factors identified in the literature as potentially predictive will be entered into multivariable logistic regression models. Final models will be determined by taking into account the final sample size for each study group, likely causal pathway, collinearity, and clinical significance. Analysis of variance (ANOVA) and generalised linear models will be used to examine the change in maternal kidney function during pregnancy. We will examine the distribution and pattern of missing data for key study and outcome factors with high proportion of missing data. Multiple imputation will be performed for variables qualified as ‘missing at random’ [42]. For variables with pattern of missing data unlikely to be ‘missing at random’, a ‘proxy’ category for missing data will be created for variables with > 2% missing and a sensitivity analysis will be conducted to explore the effect of missing data. A *p*-value < 0.05 or a confidence interval not including unity will be considered statistically significant. All statistical analyses will be performed using SPSS 24.0 software (Armonk, NY, USA: IBM Corp.).

## Discussion

This AMOSS study will highlight the issue of serious kidney disease in pregnancy in Australia, with heightened awareness in approximately 260 maternity units and among obstetric and renal healthcare providers across the country. The study will focus on cases of serious kidney disease in pregnancy that, while uncommon, can cause significant morbidity for mothers and babies, present clinical complexity for clinicians, and place a disproportionate burden on health care resources. The study will fill an important knowledge gap on the prevalence and models of care for this high-risk cohort of women.

The strengths of this study come from the AMOSS research model, which encompasses 96% of hospital births in Australia, and where cases can be identified prospectively and data collected after birth. In addition, multiple sources to identify eligible participants will increase the ability to capture cases, thereby minimising missed cases. The main challenge in this study will be the data collection for women who (1) receive their obstetric and renal care at different locations, or (2) receive renal care at a non-AMOSS site. To overcome these challenges, ethics approval was granted at most sites for the AMOSS Project Coordinator to receive minimal identifying details of prospective patients receiving renal care at one site and pass those details on to the onsite AMOSS data collector at the woman’s maternity hospital. For women receiving renal care at a non-AMOSS site, separate ethical and/or governance approval for data collection at the specialist clinic and matching with maternity data will be sought on a case-by-case basis.

The results of this study will be immediately relevant and enable translation into clinical practice. Specifically, we will.

- Improve the evidence base for pre-pregnancy counselling and pregnancy management, and will promote this as an important pathway towards improved outcomes. We will identify healthcare professional groups to target for strategic education in specialist and primary care, and mechanisms to target high-risk women antenatally. Previous AMOSS studies have led to important improvements in safety and quality of care for pregnant women suffering from rare and under-studied conditions, where there is often a lack of data and clinical experience to guide management.
- Promote clinical guideline and policy development in Australia by providing an evidence base describing current models of care, and highlighting gaps in care or risk factors for adverse outcomes. Currently, there are no Australian guidelines or policies to facilitate delivery of specialised pregnancy care to women with renal disease.
- Disseminate our findings to key stakeholders (clinical providers - medical, midwifery, nursing, allied health, government policy planners and patient/consumer groups) involved in the care of women with CKD and AKI, women with high-risk pregnancy, their babies and their families. We will develop resources for health providers and patients through engagement with renal and pregnancy consumer organisations.
- Use this study to form the foundation for developing new systems for ongoing data capture regarding maternal kidney disease, including the potential to re-purpose the data collection instrument into an ongoing registry.

Given that no other studies have explored serious maternal kidney disease in the Australian birth cohort, we anticipate the results will be of great interest to the obstetric and renal community nationally and internationally.

## Additional files

Additional file 1: General Information: Case report form is used to collect general maternal and neonatal data. (PDF 411 kb)

Additional file 2: Renal Disease in Pregnancy: Case report form is used to collect data specifically related to renal disease in pregnancy. (PDF 511 kb)

Additional file 3: Main outcome measurements: A list of the main outcome measures that will form the basis of data analysis. (DOCX 12 kb)

Additional file 4: Selected covariates: The list of variables that we expect to be associated with the outcomes. (DOCX 13 kb)

## Data Availability

Not applicable.

## Abbreviations

AKI: Acute Kidney Injury
AMOSS: Australasian Maternity Outcomes Surveillance System
BMI: Body Mass Index
CKD: Chronic Kidney Disease
eGFR: Estimated Glomerular Filtration Rate
