# Supplementary material for: Serious kidney disease in pregnancy: an Australian national cohort study protocol
Source: BMC Nephrol. 2019 Jun 25;20:230. doi: 10.1186/s12882-019-1393-z (PMC6593486; doi:10.1186/s12882-019-1393-z)
Supplement: Supplementary file 1 — General Information: Case report form is used to collect general maternal and neonatal data. (PDF 411 kb) [file 12882_2019_1393_MOESM1_ESM.pdf]

GENERAL INFORMATION ('MATERNITY SURVEY')

This paper-based form is included in your folder for your information, and in case you do not have access to the internet and need to submit a Case – *(normally you would use the web-based survey to enter data)*. If you need to use this form, please copy it first, along with the relevant condition/s.

Instructions

1. Please do not enter any personally identifiable information (e.g. name, address or hospital number) on this form: all identifying information is entered on the log sheet.
2. Enter this case on your log sheet. Record the ID on the front of this form against the woman's name in your log book (ID number obtained from web system, or contact AMOSS).
3. Fill in the form using the information available in the woman's case notes.
4. Tick the boxes as appropriate. If you require any additional space to answer a question please use the space provided at the end of this General Information or Condition form.
5. Please complete all dates in the format DD/MM/YY, unless otherwise indicated.
6. Please complete all times using the 24hr clock e.g. 14:34.
7. Definition of each variable is contained in the data dictionary (accessed at www.amoss.com.au)
8. If you encounter any problems with completing this form, please contact the AMOSS Project Coordinator amoss@uts.edu.au or use the space at end of each section to describe the problem.

Details about the person filling in this form

Name of person completing the form ____

Work phone number ______________________________

Professional group

- Midwife ☐
- Obstetrician ☐
- Obstetric physician ☐
- Maternal Fetal Medicine (MFM) specialist ☐
- Study coordinator (RHD, GBC etc) ☐
- Health information manager/Quality assurance officer ☐
- Anaesthetist ☐
- Cardiologist ☐
- Cardiac nurse ☐
- Renal physician ☐
- Renal nurse ☐
- Echotechnician/Echocardiographer ☐
- Physician/District Medical Officer (DMO) ☐
- RMO/Registrar (specify type of Registrar) ☐
- GP / GP with Obstetric Diploma ☐
- Remote area nurse (RAN) ☐
- Registered Nurse (other) ☐
- Other – please specify ☐ ______________________________

Date data form was completed ☐☐/☐☐/☐☐

Has the Medical Record Number (MRN) been recorded against the AMOSS Case ID in the Log Sheet?

Yes ☐ No ☐ (If no, please do so now. This is the only identified reference for case queries)

In the case of queries, you may find it useful to keep a copy of this form. *Don't forget to enter the Case details on the Log sheet! Contact AMOSS if you need a new one.* Thank you.

Section 1: Woman's details

1.1 Age in years ☐☐

1.2 What country was the woman born in? ______________________________________

1.3 Indigenous status

(Answer only if you are entering the data from an Australian hospital)

- Aboriginal but not Torres Strait Islander origin ☐
- Torres Strait Islander but not Aboriginal origin ☐
- Both Aboriginal and Torres Strait Islander origin ☐
- Neither Aboriginal nor Torres Strait Islander origin ☐
- Not stated/inadequately described ☐

Maori and or Pacifca identification *(Answer only if you are entering the data from an Australian hospital)*

Does the woman identify as a Maori or Pacific Islander?
(Answer this only if you are entering the data from an Australian site)
☐ No ☐ Yes - Pacific Islander ☐ Yes - Maori ☐ Not known

(Answer this only if you are entering the data from a New Zealand hospital)

- New Zealand European ☐
- Maori ☐
- Samoan ☐
- Cook Island Maori ☐
- Tongan ☐
- Niuean ☐
- Chinese ☐
- Indian ☐
- Other (please specify) ☐

____

1.4 Is English the primary language spoken at home? Yes ☐ No ☐ Not known ☐

(If not English) What is the language spoken at home?

1.5 Relationship status

- Never married ☐
- Widowed ☐
- Divorced ☐
- Separated ☐
- Married ☐
- De facto ☐
- Not stated ☐

1.6 Residential postcode ☐☐☐☐

1.7 Was the admission as a public or private patient? Private ☐ Public ☐ Not eligible for Medicare☐

1.8 Date of antenatal booking visit ___/___/____

1.9 Height at booking (cm) ☐ ☐ ☐ cm

1.10 Weight at booking (kg) ☐☐☐ kg

1.11 Smoking status

- Never smoked ☐
- Quit smoking before becoming pregnant ☐
- Quit smoking during pregnancy (<20 weeks) ☐
- Quit smoking during pregnancy (≥ 20 weeks) ☐

Continued to smoke during pregnancy ☐
Smoking at booking visit, but not known if continued during pregnancy ☐
Not smoking at booking visit, but not known if smoked during rest of pregnancy ☐
Not known ☐

Section 2: PREVIOUS pregnancies

2.1 Has the woman been pregnant before? Yes ☐ No ☐ Not known ☐
(If No or Not known please go to Section 3)

2.2 What is the woman's gravidity? ☐☐
(The total number of pregnancies, including the current one)

2.2.1 How many months since preceding birth/pregnancy termination/miscarriage? ☐☐ months
☐ Not known ☐ Not applicable *(parity = 0)*

2.3 What is the woman's parity? ☐☐
(The number of pregnancies that have resulted in a live birth or stillbirth – excluding current pregnancy)
If no previous pregnancies, *please go to section 3*

2.4 Did this woman have a prior caesarean section? Yes ☐ No ☐ Not known ☐ Not applicable *(parity = 0)* ☐
If yes, how many? ☐☐
Was the immediately preceding birth by caesarean section? Yes ☐ No ☐ Not known ☐

Section 3: BEFORE this pregnancy

3.1 Previous or pre-existing medical or obstetric problems ***(prior to this pregnancy)***

**Neurological** (Epilepsy, Migraine, Multiple sclerosis, Meningitis, Spinal cord injury, Myasthenia gravis, other)	Yes ☐ No ☐ Not known ☐	(if yes, please detail)
**Mental health** (Anxiety, Depression, Postnatal depression, Puerperal psychosis, Schizophrenia, Bipolar disorder, other)<br>**Substance use**(Methadone/ Buprenophine opiate substitution therapy, or a known user of illicit drugs, Alcohol abuse	Yes ☐ No ☐ Not known ☐<br>Yes ☐ No ☐ Not known ☐	
**Circulatory/Cardiac** (Atrial fibrillation, Endocarditis, Cardiomyopathy, Cardio-respiratory arrest, Cerebrovascular accident/stroke, Congenital heart disease with follow up in childhood, Hypertension (pre-existing), Ischaemic heart disease, Marfans, Myocardial infarction, Pulmonary oedema, Pulmonary hypertension, Rheumatic fever, Rheumatic heart disease, Thromboembolism, Valve disorder (non-RHD), other)	Yes ☐ No ☐ Not known ☐	
**Respiratory** (Asthma, Bronchiectasis, Cystic fibrosis, other)	Yes ☐ No ☐ Not known ☐	
**Gastrointestinal** (Gastric banding/stomach stapling, Fatty liver disease, Cholecystitis, Crohns Disease, Ulcerative colitis, Inflammatory bowel disease, other)	Yes ☐ No ☐ Not known ☐	
**Renal** (Recurrent UTIs,	Yes ☐ No ☐ Not known ☐	

	Glomerulonephritis, dialysis, renal transplant, other)<br>*(if yes)* What was the creatinine level at the booking visit?		Creatinine level (mmol) ________
	**Endocrine/Nutritional/Metabolic** (Diabetes mellitus Type I or Diabetes mellitus II [pre-existing], Phaeochromocytoma, other)	Yes ☐ No ☐<br>Not known ☐	
	**Haematological** (Nutritional anaemia, pulmonary embolism, deep vein thrombosis, other)	Yes ☐ No ☐<br>Not known ☐	
	**Musculoskeletal and connective** Systemic lupus erythematosus (SLE), other	Yes ☐ No ☐<br>Not known ☐	
	**Neoplasms** (cervical cancer, gestational trophoblastic tumour, gestational breast cancer, Indicate type and if current or previous)	Yes ☐ No ☐<br>Not known ☐	
	**Infections** (Influenza A, Streptococcus A , Hepatitis, Acute rheumatic fever, Syphilis, HIV, Tuberculosis, other)	Yes ☐ No ☐<br>Not known ☐	
	**Obstetric** (Gestational diabetes mellitus, Gestational trophoblastic tumour, Gestational hypertension, Pre-eclampsia, Eclampsia, Chorioamnionitis, Breech, premature rupture of membranes, Retained placenta, Obstetric tear (3rd or 4th degree), Uterine rupture, amniotic fluid embolism, pulmonary embolism, Retained placenta, Placenta accrete or other placental abnormalities, Primary or secondary haemorrhage (more than 1000 mls), Vasa praevia, other)	Yes ☐ No ☐<br>Not known ☐	
	**Fetal and newborn** (Antepartum stillbirth, Intrapartum stillbirth, Stillbirth not specified, Neonatal death, Congenital anomaly, Intra-uterine growth restriction, other)	Yes ☐ No ☐<br>Not known ☐	
	**Any other previous or pre-existing medical or obstetric problems not included above?** *(please specify)*<br>____		

Sections 4 & 5: THIS pregnancy

4.1 What is the estimated date of birth? (EDB / EDC) ☐☐/☐☐/☐☐

4.2 Was this pregnancy the result of assisted reproductive technology? Yes ☐ No ☐ Not known ☐

4.3 Was this a multiple pregnancy? Yes ☐ No ☐ Not known ☐

If yes, please specify the number of fetuses ☐

Model of Care

4.4 Please select the antenatal allocated model of care at the booking of pregnancy.

(Answer only if you are entering the data from an Australian hospital)

- Private obstetrician (specialist) care ☐
- Private midwifery care ☐
- General practitioner obstetrician care ☐
- Shared care ☐
- Combined care ☐

Public hospital maternity care ☐
High risk public hospital maternity care ☐
Team midwifery care ☐
Midwifery group practice caseload care ☐
Remote area care ☐
No formal care ☐
Other ☐ Specify ____

4.4 a Did this model of care change during this pregnancy? Yes ☐ No ☐ Not known ☐ *(Aust only)*

4.4.b If yes, at what gestational age? ☐ ☐ *(Aust only)*

4.4.c The model of care was changed to: *(Aust only)*

Private obstetrician (specialist) care ☐
Private midwifery care ☐
General Practitioner obstetrician care ☐
Shared care ☐
Combined care ☐
Public hospital maternity care ☐
High risk public hospital maternity care ☐
Team midwifery care ☐
Midwifery group practice caseload care ☐
Remote area care ☐
No formal care ☐
Other ☐ Specify ____

4.4 Did mother have an identified lead maternity carer (LMC) during pregnancy?

Yes ☐ No ☐ Not known ☐

(Answer this only if you are entering the data from a New Zealand hospital)

4.4a Who was the mother's Lead Maternity Carer at booking? *(NZ only)*

Self employed midwife ☐
Hospital employed midwife ☐
Hospital antenatal clinic ☐
High-risk/maternal fetal medicine ☐
General practitioner ☐
Obstetrician (private) ☐
Other ☐ - Specify ____

4.4b Who was the mother's Lead Maternity Carer at birth? *(NZ only)*

Self employed midwife ☐
Hospital employed midwife ☐
Hospital antenatal clinic ☐
High-risk/maternal fetal medicine ☐
General practitioner ☐
Obstetrician (private) ☐
Not applicable ☐
Other ☐ -

4.4c Specify details if the Lead Maternity Carer not listed, and indicate whether at booking or birth (or both). If not relevant enter N/A

____

Transfer

4.5	Was the woman transferred during antenatal period?	Yes ☐ No ☐ Not known ☐
4.5a	*(If yes)* Was she transferred TO your hospital from another facility?	Yes ☐ No ☐ Not known ☐
4.5b	Was she transferred FROM your hospital?	Yes ☐ No ☐ Not known ☐
4.5c	At what gestational age was the woman transferred?	☐ ☐ weeks

4.5d What was the *main* reason for the transfer?

Identified high-risk

Hospital protocol

After diagnosis of the condition being studied

Preempt complications resulting from the condition being studied

Access to facilities (eg specialist cardiac or oncology)

Other ☐ - Specify ____

Not known

4.5e Details of transfer (specify reason for transfer if not listed above, and give details related to transfer)

4.6 Are all women transferred out of their community to give birth? Yes ☐ No ☐ Not known ☐

Medication

4.7 Did the woman use any prescribed medication during the pregnancy? Yes ☐ No ☐ Not known ☐
If yes, please list ____

4.8 Were there any prescribed medication ceased because of this pregnancy? Yes ☐ No ☐ Not known ☐

If yes, please list ____

4.9 Was the woman immunised against Influenza? Yes ☐ No ☐ Not known ☐

Section 5 This Pregnancy

5.1 Obstetric/medical problems during ***this*** pregnancy (before birth)?

**Neurological** Epilepsy, Multiple sclerosis, Myasthenia gravis, other	Yes ☐ No ☐ Not known ☐	(if yes, please detail including date/s and management)
**Mental health** (eg Anxiety, Depression, Puerperal psychosis, Schizophrenia, Bipolar disorder, other)<br>**Substance use:** ((Methadone/ Buprenophine opiate substitution therapy, or a known user of illicit drugs during pregnancy; Alcohol abuse	Yes ☐ No ☐ Not known ☐<br><br>Yes ☐ No ☐ Not known ☐	
**Circulatory/Cardiac** (Atrial fibrillation, Endocarditis, Cardiomyopathy, Cardio-respiratory arrest, Cerebrovascular accident/stroke, Essential hypertension, Congenital heart disease, Ischaemic heart disease, Marfans, Myocardial infarction, Pulmonary oedema, Pulmonary hypertension, Rheumatic fever, Rheumatic heart disease, Thromboembolism, Valve disorder (non-RHD), other)	Yes ☐ No ☐ Not known ☐	
**Respiratory** (Asthma, Bronchiectasis, Cystic fibrosis, other)	Yes ☐ No ☐ Not known ☐	

**Gastrointestinal** (Gastric banding/stomach stapling, Fatty liver disease, Cholecystitis, Crohns Disease, Ulcerative colitis, Inflammatory bowel disease, other)	Yes ☐ No ☐<br>Not known ☐	
**Renal** (Recurrent UTIs, Glomerulonephritis, dialysis, renal transplant, other)<br>*(if yes)* What was the creatinine level at the booking visit?	Yes ☐ No ☐<br>Not known ☐<br><br>Creatinine level (mmol)	______________
**Endocrine/Nutritional/Metabolic** (Diabetes mellitus Type I or II [pre-existing], Phaeochromocytoma, other)	Yes ☐ No ☐<br>Not known ☐	
**Haematological** (Nutritional anaemia, Pulmonary embolism, Deep vein thrombosis, Thrombocytopenia, other)	Yes ☐ No ☐<br>Not known ☐	
**Musculoskeletal and connective** Systemic lupus erythematosus (SLE), other	Yes ☐ No ☐<br>Not known ☐	
**Neoplasms** (Cervical cancer, Gestational breast cancer<br>(Indicate type and if diagnoses during this pregnancy or recurrence), other	Yes ☐ No ☐<br>Not known ☐	
**Infections** Influenza A, Streptococcus A or B, Hepatitis, Acute rheumatic fever, Syphilis, HIV/AIDS, Tuberculosis, Listeria, Toxoplasmosis, Cytomegalovirus(CMV), other	Yes ☐ No ☐<br>Not known ☐	
**Obstetric** (Gestational diabetes mellitus, Gestational hypertension, Pre-eclampsia, Eclampsia, Antepartum haemorrhage, Threatened premature labour,<br>Placenta accrete or other placental abnormalities, Vasa praevia, Chorioamnionitis, Antenatal pulmonary embolism, Other – give details)	Yes ☐ No ☐<br>Not known ☐	
**Surgery during pregnancy?**	Yes ☐ No ☐<br>Not known ☐	

Other *(please specify)*

____

Give details of diagnosis, management and outcomes

5.2 Was the woman admitted to antenatal ward, ICU, CCU or HDU for higher care during pregnancy (before birth)?

No ☐

Yes, antenatal ward or higher dependency unit ☐

Yes, ICU ☐

Yes, Coronary Care Unit (CCU) ☐

Yes, Antenatal and ICU ☐

Yes, Antenatal and CCU ☐

Yes, ICU and CCU ☐

Other (specify) ☐

For each admission, state admission and discharge dates, indication, and treatment given.

5.2b Gestation at admission ☐☐ weeks (if multiple admissions, indicate here) ______________________________

5.2c Total number of days ☐ ☐ days (in any unit)

Not known ☐

Please specify reason and management____

5.3 What is the current status of this pregnancy?

Given birth ☐ Still pregnant ☐

Section 6: Labour and birth details

6.1 Did the woman labour? Yes ☐ No ☐ Not known ☐

If yes, please state date and time of onset of labour ☐☐/☐☐/☐☐ ☐☐:☐☐

If no, go to 2.2

6.1a	Was labour induced?	Yes ☐ No ☐ Not known ☐
6.1b	Was labour augmented?	Yes ☐ No ☐ Not known ☐
6.1c	Did the membranes rupture spontaneously?	Yes ☐ No ☐ Not known ☐
6.1d	Was syntocinon used during labour?	Yes ☐ No ☐ Not known ☐
6.1e	Was a prostaglandin used before or during labour?	Yes ☐ No ☐ Not known ☐

6.1f Length of labour 1st stage ☐☐:☐☐ 2nd stage ☐☐:☐☐ 3rd stage ☐☐:☐☐

6.2 Was birth by caesarean section? Yes ☐ No ☐ Not known ☐

6.2a If yes, please state the urgency of caesarean section

RANZCOG Category 1 ☐
RANZCOG Category 2 ☐
RANZCOG Category 3 ☐
RANZCOG Category 4 ☐
Not Known ☐

6.2b Please give the indication for caesarean section

Obstetric indication ☐

Not relevant: no obstetric indication ☐
Poor progress in labour ☐
Delay in second stage ☐
Failure to establish labour ☐
High presenting part ☐
Hypertension ☐
Poor descent ☐
Cord prolapse/presentation ☐
Placenta praevia ☐
Abruption placenta ☐

Antepartum or intrapartum haemorrhage ☐
Vasa praevia ☐
Previous caesarean section ☐
Failed instrumental delivery ☐
Not known ☐

Maternal indication ☐
Not relevant: no maternal indication ☐
Previous uterine surgery (excluding C/S) ☐
Active genital herpes ☐
Uterine anomaly ☐
Fibroid/ pelvic mass stenosis ☐
Maternal disease/surgery related to the condition being studied ☐
Maternal disease/surgery not related to the condition being studied ☐
Maternal request ☐
Maternal exhaustion ☐
Not known ☐

Fetal indication ☐
Not relevant: no fetal indication ☐
Non-reassuring FHR trace ☐
Non-reassuring FBS ☐
Abnormal fetal welfare studies ☐
Fetal growth restriction ☐
Breech/face/brow presentation ☐
Oblique/transverse lie ☐
Multiple fetus ☐
Fetal abnormality ☐
Not known ☐

Other indication Yes ☐ No ☐ Not known ☐

If yes, please specify ____

6.3 Please indicate any analgesia or anaesthesia used during labour and birth

Nil ☐
Nitrous oxide ☐
Systemic opioids ☐
Pudendal or caudal block ☐
Epidural ☐
Spinal ☐
Combined spinal – epidural ☐
General anaesthesia ☐
Other ☐

____

Section 7: Maternal Outcomes

7.1 Did the woman experience a major postpartum haemorrhage? (estimated blood loss > 1000ml) Yes ☐ No ☐ Not known ☐

If yes:

At Birth ☐

During the first 24 hours after birth (primary postpartum haemorrhage) ☐ After 24 hours postpartum (secondary postpartum haemorrhage) ☐

Not Known ☐

7.1a *(If yes)* What was the estimated blood loss? _____________ ml

7.1b Did the woman have blood products because of this postpartum haemorrhage? Yes ☐ No ☐ Not known ☐

If yes, how many units of blood products did the woman have? ☐☐

7.1c

What treatment(s) were used to control bleeding? **Uterotonic interventions:**

Syntocinon infusion Yes ☐ No☐ Not known ☐

Syntometrine Yes ☐ No☐ Not known ☐

Prostaglandin F2α Yes ☐ No☐ Not known ☐

Misoprostol Yes ☐ No☐ Not known ☐Tranexamic acid Yes ☐ No☐ Not known ☐

Transfusion support:

Recombinant Factor VIIa Yes ☐ No☐ Not known ☐

Fibrinogen concentrate Yes ☐ No☐ Not known ☐

Prothrombin complex concentrate Yes ☐ No☐ Not known ☐

Surgical/radiological interventions:

Suturing of perineal tears/lower genital tract trauma Yes ☐ No☐ Not known ☐

Examination under anaesthesia and manual removal of placenta/retained products Yes ☐ No☐ Not known ☐

Intra-abdominal packing Yes ☐ No☐ Not known ☐

Intra-uterine balloon Yes ☐ No☐ Not known ☐

Intra-uterine packing Yes ☐ No☐ Not known ☐

Vessel embolization (radiological) Yes ☐ No☐ Not known ☐

Vessel ligation Yes ☐ No☐ Not known ☐

Intra-arterial balloons (radiology – guided) Yes ☐ No☐ Not known ☐

B-Lynch or other brace sutures Yes ☐ No☐ Not known ☐

Other Yes ☐ No☐ Not known ☐

If other, please specify ______________________________________

____

7.2 Did the woman have a hysterectomy? Yes ☐ No ☐ Not known ☐

7.3-7.7 Did any of the following morbidities occur?

**Other Obstetric** (Eclampsia, Prolonged rupture of membranes, Shoulder dystocia, Retained placenta, Obstetric 3rd or 4th degree tear, Uterine rupture, Amniotic embolism, Puerperal sepsis, Placenta accrete or other placental abnormalities, other)	Yes ☐ No ☐ Not known ☐
**Cardiac/circulatory** (Atrial fibrillation, Endocarditis, Cardiomyopathy, Cardio respiratory arrest, Cerebrovascular accident/ stroke, Essential hypertension, Intracerebral bleed, Ischaemic heart disease, Myocardial infarction, Peripartum cardiomyopathy, Pulmonary oedema, Pulmonary hypertension, Rheumatic fever, Rheumatic heart disease, Thromboembolism, Valve disorder (non-RHD), other)	Yes ☐ No ☐ Not known ☐
**Mental health** (Puerperal psychosis, Withdrawal from opiates or sedatives, Withdrawal from alcohol, other)	Yes ☐ No ☐ Not known ☐
**Infections** (MRSA, Wound infection, other)	Yes ☐ No ☐ Not known ☐

Other *(please specify)* ____

Give details of diagnosis, management and outcomes

7.8 Did the woman go/return to operating theatre within 48 hours of giving birth? Yes ☐ No ☐ Not known ☐

(If yes) Give details ______________________________________

7.9 Was the woman admitted to ICU? Yes ☐ No ☐ Not known ☐ (If yes) How many days? _________

7.10 Was the woman admitted to Cardiac Care Unit (CCU)?

Yes ☐ No ☐ Not known ☐ (If yes) How many days? _________

7.11 Was the woman admitted to High Dependency Unit (HDU)? Yes ☐ No ☐ Not known ☐ (If yes) How many days? _________

7.12 Was the woman managed in BS (Birthing suite) (or other unit that provides additional care and monitoring) postpartum? Yes ☐ No ☐ Not known ☐

Separation status

7.13 Was the woman transferred to/from your hospital *during labour*? Yes ☐ No ☐ Not known ☐

(If yes) Transferred *from* our hospital? Yes ☐ No ☐ Not known ☐
Transferred *to* our hospital? Yes ☐ No ☐ Not known ☐
Transferred TO our hospital, then transferred back to the original hospital Yes ☐ No ☐ Not known ☐
Transferred TO our hospital, then transferred to another hospital Yes ☐ No ☐ Not known ☐

(If yes) Reason for transfer:
Maternal or fetal complication not related to the condition being studied ☐
Due to diagnosis of the condition being studied ☐
Not known ☐
Other/give details ____

7.14 Was the woman discharged home from hospital Yes ☐ No ☐ Not known ☐

7.15 Was the woman transferred to/from your hospital *postpartum*? Yes ☐ No ☐ Not known ☐
(If yes) Transferred *from* our hospital? Yes ☐ No ☐ Not known ☐
Transferred *to* our hospital? Yes ☐ No ☐ Not known ☐
Transferred TO our hospital, then transferred back to the original hospital Yes ☐ No ☐ Not known ☐
Transferred TO our hospital, then transferred to another hospital Yes ☐ No ☐ Not known ☐

(If yes) What was the *main* reason for transfer:

Return to local hospital/community ☐
Maternal complications not related to the condition being studied ☐
Maternal complication related to the condition being studied ☐
Accompany baby being transferred to a higher level care hospital ☐
Woman's choice ☐
Not known ☐
Other ☐

If other, please specify ____

7.16 What was the *total* length of stay in the hospital? _________days

7.17 Did the woman die? Yes ☐ No ☐ Not known ☐

7.17a If yes, please specify date of death ☐☐/☐☐/☐☐

7.17b What was the primary cause of death, as stated on the death certificate?

____

7.17c Was a postmortem examination undertaken? Yes ☐ No ☐ Not known ☐

Section 8: Infant Outcomes *Please print out another copy of page for each infant if more than one:*

8.1 Date and time of birth ☐☐/☐☐/☐☐ ☐☐:☐☐

8.2 **Place of birth *(select one):***

Birth suite ☐
Operating theatre ☐
Intensive care unit ☐
Cardiac care unit ☐
Emergency department ☐
Other (detail) ____

8.3 **Mode of birth**

Unassisted vaginal birth (vertex) ☐
Forceps ☐
Vacuum extraction ☐
Caesarean section ☐
Vaginal breech ☐
- Frank ☐
- Complete ☐
- Footling ☐
- Kneeling (incomplete) ☐
- Not known ☐

(If breech)

Did the woman have ECV (External cephalic version)? Yes☐ No☐ Not known ☐
ECV available and accepted ☐ ECV not available ☐
ECV contraindicated ☐
ECV available not offered ☐
ECV available declined ☐
Not known ☐

8.4 Gender Male ☐ Female ☐ Indeterminate ☐ Not stated ☐

8.5 Birth gestation (completed weeks) ☐☐ weeks

8.6 Birth weight (g) ☐☐☐☐ grams

8.7 What was the birth order of this neonate?

- Singleton or first birth of a multiple birth ☐
- Second of a multiple birth ☐
- Third of a multiple birth ☐
- Fourth of a multiple birth ☐
- Fifth of a multiple birth ☐
- Sixth of a multiple birth ☐
- Other ☐
- Not stated ☐

8.8 What was the infant's condition at birth?

- Live born ☐
- Ante-partum Stillborn ☐
- Intra-partum Stillborn ☐
- Stillborn (not specified) ☐

(If stillborn, go to question 8.8)

- Not known ☐

8.8a What was the 5 minute Apgar score? ☐

8.8b-c Was the infant admitted to the Neonatal intensive care unit or Special care nursery?

- Neonatal intensive care unit Yes ☐ No ☐ Not known ☐
- Special care nursery Yes ☐ No ☐ Not known ☐

8.8d Did the infant require resuscitation measures?

- No ☐
- Oxygen ☐
- Neopuff or CPAP mask only ☐
- Suction ☐
- Neopuff or CPAP mask + Suction + Oxygen ☐
- Intubated with CPAP only
- Intubated with IPP (intermittent positive pressure)
- IPP intubation + Cardiac compression ☐
- Cardiac compression ☐
- Resuscitation drugs (nalaxone, adrenaline etc) ☐
- Resuscitation drugs + IPP intubation + Cardiac compression ☐
- All of the above ☐
- Other ☐
- Not known ☐

8.8e Did the infant require any further respiratory support?

- No ☐
- High flow nasal cannula ☐
- CPAP mask only ☐
- Intubated with CPAP only ☐
- Intubated with IPP (intermittent positive pressure ventilation) ☐
- IMV (intermittent mandatory ventilation) ☐
- HFOV (high frequency oscillatory ventilation) ☐
- Other ☐

8.8e Did any major infant complications occur? Yes ☐ No ☐ Not known ☐

If yes, please specify

____

8.9 Was the baby born with any congenital malformations? Yes ☐ No ☐ Not known ☐

If yes, please specify

____

8.10 What was the separation status of the infant?

Discharged home ☐
Transferred to another health facility ☐
Died ☐

8.10a If the infant died, please specify the date of death ☐☐/☐☐/☐☐

8.10b What was the primary cause of death, as stated on the death certificate?

____

8.10c Was an autopsy performed? Yes ☐ No ☐ Not known ☐

8.11 Any other information?

Please use this space to enter any other information you feel may be important

____

____

____

____

____

____

____

____

____

____

____

(Now complete the condition specific survey/s. Thanks!)
