# Supplementary material for: Serious kidney disease in pregnancy: an Australian national cohort study protocol
Source: BMC Nephrol. 2019 Jun 25;20:230. doi: 10.1186/s12882-019-1393-z (PMC6593486; doi:10.1186/s12882-019-1393-z)
Supplement: Supplementary file 2 — Renal Disease in Pregnancy: Case report form is used to collect data specifically related to renal disease in pregnancy. (PDF 511 kb) [file 12882_2019_1393_MOESM2_ESM.pdf]

AMOSS Case ID ___________________________

Case Report Form

RENAL DISEASE IN PREGNANCY

Details about the person filling in this form

Name of person completing the form ____
Work phone number ________________
Professional group

- Midwife ☐
- Obstetrician ☐
- Obstetric physician ☐
- Maternal-fetal specialist ☐
- AMOSS study coordinator ☐
- Health information manager/Quality assurance officer ☐
- Anaesthetist ☐
- Renal physician ☐
- Renal nurse ☐
- Physician/District Medical Officer (DMO) ☐
- RMO/Registrar ☐
- GP / GP with Obstetric Diploma ☐
- Remote area nurse (RAN) ☐
- Registered Nurse (other) ☐
- Other – please specify ☐ ______________________________

Date data form was completed ☐☐/☐☐/☐☐

Has the Medical Record Number (MRN) been recorded against the AMOSS Case ID in the Log Sheet?
Yes ☐ No ☐ (*If no, please do so now. This is to help you easily identify the patient, if AMOSS has any questions about this case in the future.)*

Inclusion criteria

All women in Australia who present with advanced chronic kidney disease or severe acute kidney injury before or during pregnancy.

Reporting guidelines
Inclusion:
If the woman has any of the following:

1. A working kidney transplant (all women regardless of kidney transplant function) Yes ☐ No ☐
2. Receiving any long-term dialysis before conception and continuing any dialysis during this pregnancy Yes ☐ No ☐
3. Started any dialysis during this pregnancy (any dialysis - either once off, temporary or permanent dialysis) Yes ☐ No ☐
4. Known pre-conception eGFR <45ml/min/1.73m2 (known to have a serum creatinine >130-150 µmol/L BEFORE conception, regardless of the serum creatinine reading during pregnancy; eGFR is reported along with serum creatinine in the biochemistry report) Yes ☐ No ☐
5. Newly identified kidney impairment with any serum creatinine reading of >150 µmol/L on 2 readings at least 24 hours apart during this pregnancy Yes ☐ No ☐

Please contact AMOSS amoss@uts.edu.au or 02 9514 8041 with any queries about inclusion.

SECTION 1: GENERAL RENAL HISTORY

1. Did this woman receive care in a maternity service co-located with a renal service?
Yes ☐ No ☐ Not known ☐

1.1. *(If yes)* Was the primary case medical record shared between these services?
Yes ☐ No ☐ Not known ☐

2. Renal History Prior to Pregnancy *(where more than one criterion applies, choose all that apply)*

**2.1.**	**Functioning kidney transplant**	Yes ☐ No ☐
**2.2.**	**Chronic maintenance dialysis**	Yes ☐ No ☐
**2.3.**	**Known kidney impairment or kidney disease before this pregnancy**	Yes ☐ No ☐
**2.4.**	**No known renal history – kidney impairment was diagnosed for the first time during this pregnancy**	Yes ☐ No ☐

3. Underlying cause or condition causing kidney disease in this woman *(choose all that apply)*

**3.1.**	**Glomerulonephritis**	Yes ☐ No ☐	
	(If yes) Lupus nephritis		Yes ☐ No ☐
	IgA nephropathy		Yes ☐ No ☐
	Vasculitis		Yes ☐ No ☐
	Minimal change disease		Yes ☐ No ☐
	Focal Segmental Glomerulosclerosis		Yes ☐ No ☐
	Membranous nephritis		Yes ☐ No ☐
	Anti-GBM disease		Yes ☐ No ☐
	Other glomerulonephritis		Yes ☐ No ☐
	(If yes) specify ______________________________		
**3.2.**	**Reflux nephropathy**	Yes ☐ No ☐	
**3.3.**	**Genetic disease**	Yes ☐ No ☐	
	(If yes) Hereditary nephritis (Alport's disease or other)		Yes ☐ No ☐
	Polycystic kidney disease		Yes ☐ No ☐
	Other genetic disease		Yes ☐ No ☐
	(If yes) specify ______________________________		
**3.4.**	**Congenital renal disease** *(e.g.congenital renal obstruction, congenital malformation, obstructive uropathy)*	Yes ☐ No ☐	
**3.5.**	**Diabetic nephropathy**	Yes ☐ No ☐	
**3.6.**	**Hypertension**	Yes ☐ No ☐	
**3.7.**	**Kidney stones**	Yes ☐ No ☐	
**3.8.**	**Interstitial nephritis**	Yes ☐ No ☐	
**3.9.**	**Obstetric complications during this pregnancy**	Yes ☐ No ☐	
	(If yes) Preeclampsia		Yes ☐ No ☐
	Placental abruption		Yes ☐ No ☐
	Hyperemesis gravidarum		Yes ☐ No ☐
	Ovarian hyperstimulation syndrome (OHSS)		Yes ☐ No ☐
	Amniotic fluid embolism		Yes ☐ No ☐
	Other Obstetric complications		Yes ☐ No ☐
	(If yes) specify ______________________________		
**3.10.**	**Haemolytic uraemic syndrome**	Yes ☐ No ☐	
**3.11.**	**Cortical necrosis**	Yes ☐ No ☐	
**3.12.**	**Acute fatty liver of pregnancy**	Yes ☐ No ☐	

3.13. **Sepsis** *(Renal sepsis, Non-renal sepsis, septic shock)* Yes ☐ No ☐

(If yes) Renal sepsis (inc pyelonephritis, TB, abscess) Yes ☐ No ☐

Non-renal sepsis (including abortion) Yes ☐ No ☐

Sepsis causing septic shock Yes ☐ No ☐

Cause of sepsis ___________________________________

3.14. **Blood loss** Yes ☐ No ☐

(*If yes)* choose all that apply

Antepartum haemorrhage Yes ☐ No ☐

Post-partum haemorrhage Yes ☐ No ☐

Gastrointestinal bleed Yes ☐ No ☐

Trauma Yes ☐ No ☐

Other Yes ☐ No ☐

(If yes) specify ___________________________________

3.15. **Fluid losses** Yes ☐ No ☐

(*If yes)* choose all that apply

Vomiting or diarrhoea Yes ☐ No ☐

Polyuria Yes ☐ No ☐

Nasogastric losses Yes ☐ No ☐

Other Yes ☐ No ☐

(*If yes)* specify __________________________________

3.16. **Poor fluid intake causing dehydration** Yes ☐ No ☐

3.17. **Cardiac event** Yes ☐ No ☐

(*If yes)* choose all that apply

Myocardial infarction Yes ☐ No ☐

Heart failure Yes ☐ No ☐

Other cardiac event Yes ☐ No ☐

(If yes) specify ______________________

3.18. **Nephrotoxic drugs** Yes ☐ No ☐

(If yes) specify _____________________________________

3.19. **Mechanical obstruction of ureters or bladder** Yes ☐ No ☐

3.20. **Other** Yes ☐ No ☐

(If yes) specify ________________________________

3.21. **Underlying cause is not known (at time of survey completion)** Yes ☐ No ☐

4. **Did this woman have any kidney problems associated with previous pregnancies?**

Yes ☐ No ☐ Not known ☐ Not applicable ☐

4.1. *(If yes)* Chronic kidney disease Yes ☐ No ☐ Not known ☐

4.2. Dialysis Yes ☐ No ☐ Not known ☐

4.3. Transplant Yes ☐ No ☐ Not known ☐

4.4. Acute kidney failure Yes ☐ No ☐ Not known ☐

5. **Did this woman have a natural/native kidney biopsy PRIOR to this pregnancy?**

Yes ☐ No ☐ Not known ☐

(*If yes)* please attach non identifiable reports

6. **Did this woman have a natural/native kidney biopsy DURING this pregnancy?**

Yes ☐ No ☐ Not known ☐

(*If yes)* please attach non identifiable reports

7. **Has this woman been reviewed in a Renal Unit or by a Renal Physician?**
Yes ☐ No ☐ Not known ☐

7.1. *(If yes)* Reviewed prior to this pregnancy? Yes ☐ No ☐ Not known ☐
7.2. Reviewed during this pregnancy? Yes ☐ No ☐ Not known ☐
(If yes) Gestation of first nephrologist review (weeks) _______________
Date of review: ___/___/_____
Reviewed in a high risk pregnancy clinic Yes ☐ No ☐ Not known ☐
Reviewed in a general renal clinic Yes ☐ No ☐ Not known ☐

8. **Where was the kidney condition primarily managed?** *(select one)*
☐ Adult obstetric HDU/ICU
☐ Adult non-obstetric HDU/ICU
☐ Renal Unit
☐ General Maternity Ward
☐ Emergency Department
☐ Other
(If other) specify ____

9. **Was this woman transferred to another hospital at any time?**
Yes ☐ No ☐ Not known ☐
(*If yes)*

9.1. To access renal services Yes ☐ No ☐ Not known ☐
9.2. To access adult ICU Yes ☐ No ☐ Not known ☐
9.3. To access neonatal ICU Yes ☐ No ☐ Not known ☐

10. **Management of kidney problem** ***(choose all that apply)***

10.1. Oral Fluid therapy Yes ☐ No ☐ Not known ☐
10.2. Intravenous Fluid therapy Yes ☐ No ☐ Not known ☐
10.3. Inotropic support Yes ☐ No ☐ Not known ☐
10.4. Dialysis (complete dialysis section) Yes ☐ No ☐ Not known ☐
10.5. Antibiotics for sepsis Yes ☐ No ☐ Not known ☐
10.6. Blood transfusion Yes ☐ No ☐ Not known ☐
10.7. Immunosuppressive therapy Yes ☐ No ☐ Not known ☐
(If yes) specify _______________________

10.8. Plasma exchange Yes ☐ No ☐ Not known ☐
10.9. Eculizumab (for atypical Haemolytic uraemic syndrome) Yes ☐ No ☐ Not known ☐
10.10. Urinary catheterisation Yes ☐ No ☐ Not known ☐
10.11. Other measures to relieve urinary tract obstruction
(e.g nephrostomy, stone retrieval) Yes ☐ No ☐ Not known ☐
10.12. Diuretic therapy Yes ☐ No ☐ Not known ☐
10.13. Treatment of hyperkalaemia Yes ☐ No ☐ Not known ☐
(If yes) Dialysis for hyperkalaemia Yes ☐ No ☐ Not known ☐
10.14. Other treatments Yes ☐ No ☐ Not known ☐
(If yes) specify ____________________

11. RENAL PARAMETER READINGS IN THIS PREGNANCY – HIGHEST VALUE (*except haemoglobin*)

	**PRE-PREGNANCY**		**AT BOOKING**		**1ST TRIMESTER**		**2ND TRIMESTER**	
	Value	***Date***	***Value***	***Date***	***Value***	***Date***	***Value***	***Date***
**Serum Creatinine (μmol/L)**								
**Estimated GFR**								
**Serum Urea (mmol/L)**								
**Serum potassium (mmol/L)**								
**Serum phosphate (mmol/L)**								
**Serum uric acid (μmol/L)**								
**Haemoglobin (lowest)* (g/L)**								
**Urine Protein (P:Cr) (mg/mmol)**								
**Urine Protein (24 hour) (g/24hr)**								
**Urine A:Cr (mg/mmol)**								

	**3RD TRIMESTER**		**AT DELIVERY**		**AT DISCHARGE**	
	Value	***Date***	***Value***	***Date***	***Value***	***Date***
**Serum Creatinine (μmol/L)**						
**Estimated GFR**						
**Serum Urea (mmol/L)**						
**Serum potassium (mmol/L)**						
**Serum phosphate (mmol/L)**						
**Serum uric acid (μmol/L)**						
**Haemoglobin (lowest)* (g/L)**						
**Urine Protein (P:Cr) (mg/mmol)**						
**Urine Protein (24 hour) (g/24hr)**						
**Urine A:Cr (mg/mmol)**						

12. Was this woman admitted to hospital during this pregnancy (other than for delivery)?

Yes ☐ No ☐ Not known ☐

If yes

State admission and discharge dates, reason, and treatment given.

Date admitted ____________ Date discharged ____

Reason ____

____

Treatment ____

____

____

____

SECTION 2: TRANSPLANT HISTORY

1. **Did this woman have a functioning kidney transplant at conception?**
Yes ☐ No ☐ Not known ☐ ***IF NO, SKIP TO SECTION 3***

(If yes) **Date of current transplant** ___/___/_____

Transplant source (current transplant) *(choose one)* ☐ Living donor
☐ Deceased donor
☐ Not known

1.1. **Has this woman had more than one kidney transplant?** Yes ☐ No ☐ Not known ☐
(If yes) Total number of kidney transplants ___________

1.2. **Has this woman had a non-kidney transplant?**
Yes ☐ No ☐ Not known ☐
(If yes) Pancreas Yes ☐ No ☐ Not known ☐
Date ___/___/_____

Liver Yes ☐ No ☐ Not known ☐
Date ___/___/_____

Other Yes ☐ No ☐ Not known ☐
Date ___/___/_____
Other transplant *(specify)* ______________________________________

2. **Has this woman had a transplant kidney biopsy in the 12 months PRIOR to this pregnancy?**
Yes ☐ No ☐ Not known ☐
(If yes) attach deidentified reports

3. **Did this woman have a transplant kidney biopsy DURING this pregnancy?**
Yes ☐ No ☐ Not known ☐
(If yes) attach deidentified reports

4. **Was there treatment for transplant rejection in this pregnancy?**
Yes ☐ No ☐ Not known ☐
(If yes) How was rejection treated?

**4.1.**	Increased dose of existing immunosuppression	Yes ☐ No ☐ Not known ☐
**4.2.**	Intravenous methylprednisolone	Yes ☐ No ☐ Not known ☐
**4.3.**	T-cell depletion therapy (drugs such as ATG or OKT3)	Yes ☐ No ☐ Not known ☐
**4.4.**	Plasma exchange	Yes ☐ No ☐ Not known ☐
**4.5.**	Photopheresis	Yes ☐ No ☐ Not known ☐
**4.6.**	Other therapy	Yes ☐ No ☐ Not known ☐

Other *(specify)* ______________________________

SECTION 3: DIALYSIS IN THIS PREGNANCY

1. **Did this woman receive ANY dialysis during this pregnancy?**
Yes ☐ No ☐ Not known ☐ ***IF NO, SKIP TO SECTION 4***

(If yes) **please contact the AMOSS team if you need assistance**

2. COMMENCEMENT OF DIALYSIS IN THIS PREGNANCY (women not previously receiving dialysis)

2.1. **Did this woman commence dialysis therapy FOR THE FIRST TIME DURING this pregnancy?**
Yes ☐ No ☐ Not known ☐

IF NO, SKIP TO QUESTION 3
(If not known), ***please contact the AMOSS team for assistance***

(If yes) please answer questions below

2.2. **Reason for commencing dialysis therapy (choose all that apply)**

Newly diagnosed significant kidney dysfunction Yes ☐ No ☐ Not known ☐
Pre-existing kidney disease / impairment that became worse during pregnancy Yes ☐ No ☐ Not known ☐
Uncontrolled hypertension Yes ☐ No ☐ Not known ☐
Poor fetal growth Yes ☐ No ☐ Not known ☐
Fluid overload Yes ☐ No ☐ Not known ☐
Pre-eclampsia Yes ☐ No ☐ Not known ☐
High potassium Yes ☐ No ☐ Not known ☐
Acidosis Yes ☐ No ☐ Not known ☐
Uraemic symptoms (nausea, vomiting, itching) Yes ☐ No ☐ Not known ☐
Other Yes ☐ No ☐ Not known ☐
(If yes) specify ____

2.3. **First Haemodialysis This Pregnancy – Treatment Details**

What date did dialysis start? ____/____/_____
Dialysis duration (hours) ___________________
Type of dialysis *(choose one)*
☐ Intermittent
☐ Continuous
Heparin or citrate anticoagulation Yes ☐ No ☐ Not known ☐
Pre-dialysis creatinine ___________________ (µmol/L)
Pre-dialysis urea ___________________ (mmol/L)
Pre-dialysis phosphate ___________________ (mmol/L)
Pre-dialysis potassium ___________________ (mmol/L)
Pre-dialysis weight ___________________ (kg)
Site of first dialysis treatment *(choose one)*
☐ ICU
☐ Ward
☐ Outpatient Dialysis Unit

If this woman initially received continuous dialysis (in intensive care), was this later changed to intermittent dialysis? Yes ☐ No ☐ Not known ☐
(If yes)
Date of first intermittent dialysis ____/____/_____
Total number of intermittent dialysis treatments ___________________

Date of last dialysis treatment (if dialysis ceased before discharge)? ____/____/_____

Did this woman continue to have ongoing dialysis after discharge (or a plan for further dialysis)?
Yes ☐ No ☐ Not known ☐

Did this woman remain on dialysis permanently? Yes ☐ No ☐ Not known ☐
What date did dialysis eventually cease (if known)? ____/____/_____

2.4. Monitoring received during first dialysis treatment *(select all that apply)*

Fetal CTG Yes ☐ No ☐ Not known ☐
Dopplers Yes ☐ No ☐ Not known ☐
Maternal cardiac monitoring Yes ☐ No ☐ Not known ☐
Other monitoring Yes ☐ No ☐ Not known ☐
(If yes) specify ____

3. CHRONIC/ONGOING DIALYSIS

3.1. Date of commencing chronic maintenance dialysis *(pre-conception)* ____/ _____ / _______

3.2. Last pre-conception dialysis modality *(choose one)* ☐ peritoneal dialysis (PD) ☐ haemodialysis

3.3. Total hours of dialysis per week *(pre-conception)* ________________

3.4. Change of dialysis modality during pregnancy?
Yes ☐ No ☐ Not known ☐
(If yes) choose one ☐ Haemodialysis changed to peritoneal dialysis
☐ Peritoneal dialysis changed to haemodialysis
Date of change of modality ____/___/______
Reason for change of modality __________________________

3.5. Dialysis modality DURING this pregnancy *(choose one)*

☐ haemodialysis ***IF YES, SKIP TO QUESTION 4***
☐ peritoneal dialysis (PD) ***IF YES, SKIP TO QUESTION 6***

4. CHRONIC HAEMODIALYSIS DURING THIS PREGNANCY

Please skip this section and move to Section 4 if you have attached deidentified dialysis charts.

	PRE-PREGNANCY	1ST TRIMESTER (1-13 WEEKS)	2ND TRIMESTER (14-27 WEEKS)	3RD TRIMESTER (28+ WEEKS)
**Pre dialysis weight**	kg	kg	kg	kg
**Post dialysis weight**	kg	kg	kg	kg
**No. of dialysis sessions per week**				
**No. of hours per dialysis session**				
**Highest pre-dialysis urea (mmol/L)**				
**Heparin during dialysis**	Yes ☐ No ☐ Not known ☐	Yes ☐ No ☐ Not known ☐	Yes ☐ No ☐ Not known ☐	Yes ☐ No ☐ Not known ☐
**Dialysate K+ (mmol/L)**				
**Dialysate Bicarbonate (mmol/L)**				
**Dialysate Calcium (mmol/L)**				
**Erythropoietin administration**	Yes ☐ No ☐ Not known ☐	Yes ☐ No ☐ Not known ☐	Yes ☐ No ☐ Not known ☐	Yes ☐ No ☐ Not known ☐
**Iron administration**	Yes ☐ No ☐ Not known ☐	Yes ☐ No ☐ Not known ☐	Yes ☐ No ☐ Not known ☐	Yes ☐ No ☐ Not known ☐
**Site of dialysis *(choose one)***	Hospital ☐ Home ☐ Satellite Unit ☐	Hospital ☐ Home ☐ Satellite Unit ☐	Hospital ☐ Home ☐ Satellite Unit ☐	Hospital ☐ Home ☐ Satellite Unit ☐
**Nocturnal haemodialysis**	Yes ☐ No ☐ Not known ☐	Yes ☐ No ☐ Not known ☐	Yes ☐ No ☐ Not known ☐	Yes ☐ No ☐ Not known ☐

5. **Did this woman have any vascular access for dialysis during this pregnancy?**
Yes ☐ No ☐ Not known ☐

IF NO SKIP TO SECTION 4

(If yes) please answer questions below

5.1. **Did this woman have a pre-existing functioning arteriovenous fistula in situ?**
Yes ☐ No ☐ Not known ☐
(If yes) type of fistula (choose one) ☐ Native fistula
☐ Artificial graft

5.2. **Was an arteriovenous fistula created during THIS pregnancy?**
Yes ☐ No ☐ Not known ☐
If yes
Date at of creation ____/___/______
Serum creatinine at creation _____________________ (µmol/L)

5.3. **Was any other vascular access for dialysis used during this pregnancy?**
Yes ☐ No ☐ Not known ☐
(*If yes*) Temporary Catheter (Vascath) Yes ☐ No ☐ Not known ☐
(If yes) Date of insertion ____/___/______

Tunnelled Catheter (Permcath) Yes ☐ No ☐ Not known ☐
(If yes) Date of insertion ____/___/______

Access Related Complications

5.4. Sepsis / bacteraemia Yes ☐ No ☐ Not known ☐
(If yes) Date ____/___/______
Organism ________________
Antibiotics ________________
Other details ________________

5.5. Thrombosis Yes ☐ No ☐ Not known ☐
(If yes) Date ____/___/______

Other details ________________________________

5.6. Bleeding Yes ☐ No ☐ Not known ☐
5.7. Difficulty needling Yes ☐ No ☐ Not known ☐
5.8. Vessel stenosis requiring angioplasty Yes ☐ No ☐ Not known ☐
5.9. Other Yes ☐ No ☐ Not known ☐
(If yes) specify ________________________________

6. PERITONEAL DIALYSIS (PD)

6.1. **Was this woman receiving any peritoneal dialysis (PD) during DURING this pregnancy?**
Yes ☐ No ☐ Not known ☐ ***IF NO, SKIP TO SECTION 4***

(If yes) choose one ☐ Automated
☐ Ambulatory (manual)

6.2. **PD Regime in each trimester** *(% glucose, additional icodextran and number of exchanges – please contact the AMOSS team for assistance with data collection – amoss@uts.edu.au or (02) 9514 8041)*

1st Trimester Regime

____
____
____
____
____

2nd Trimester Regime

____
____
____
____
____

3rd Trimester Regime

____
____
____
____
____

6.3. Did PD peritonitis occur during this pregnancy?

Yes ☐ No ☐ Not known ☐

(*If yes*) Date ____/___/______
Organism ________________
Antibiotics ________________
Other (details) _________________

6.4. Did a PD catheter exit site infection occur during this pregnancy?

Yes ☐ No ☐ Not known ☐

(If yes) Date ____/___/______
Organism ________________
Antibiotics ________________
Other (details) _________________

6.5. Was PD adequacy measured during this pregnancy?

Yes ☐ No ☐ Not known ☐

(If yes) Date ____/___/______
Adequacy result ________________ *(free text)*

6.6. Was residual urine output measured during this pregnancy?

Yes ☐ No ☐ Not known ☐

(*If yes*) Date ____/___/______
Volume _________________ (ml)

6.7. Was a Peritoneal Equilibrium Test (PET) performed during this pregnancy?

Yes ☐ No ☐ Not known ☐

(If yes) Date ____/___/______
Result _________________

6.8. Did any other complications of Peritoneal Dialysis occur during this pregnancy?

Yes ☐ No ☐ Not known ☐

(If yes) Details ____
____

SECTION 4: MEDICATIONS AND OTHER THERAPIES

1. Anti-Hypertensive Medications PRIOR to Pregnancy

1.1. Did this woman take ACE Inhibitors *(e.g. perindopril, ramipril, enalapril, captopril)* or Angiotensin Receptor Blockers *(e.g. irbesartan, candesartan, telmisartan)* PRIOR to this pregnancy?

***(if yes)* Did use of ACE Inhibitors or Angiotensin Receptor Blockers cease PRIOR to this conception?**
Yes ☐ No ☐ Not known ☐

2. ANTI-HYPERTENSIVE MEDICATIONS

	PRE-PREGNANCY	ANTEPARTUM	INTRAPARTUM	POSTPARTUM	AT DISCHARGE
**Methyldopa *(Aldomet, Hydopa)***	Yes ☐ No ☐ Not known ☐	Yes ☐ No ☐ Not known ☐	Yes ☐ No ☐ Not known ☐	Yes ☐ No ☐ Not known ☐	Yes ☐ No ☐ Not known ☐
**Nifedipine *(Adalat, Adefin)***	Yes ☐ No ☐ Not known ☐	Yes ☐ No ☐ Not known ☐	Yes ☐ No ☐ Not known ☐	Yes ☐ No ☐ Not known ☐	Yes ☐ No ☐ Not known ☐
**Labetalol *(Presolol, Trandate)***	Yes ☐ No ☐ Not known ☐	Yes ☐ No ☐ Not known ☐	Yes ☐ No ☐ Not known ☐	Yes ☐ No ☐ Not known ☐	Yes ☐ No ☐ Not known ☐
**Oxprenolol (*Corbeton)***	Yes ☐ No ☐ Not known ☐	Yes ☐ No ☐ Not known ☐	Yes ☐ No ☐ Not known ☐	Yes ☐ No ☐ Not known ☐	Yes ☐ No ☐ Not known ☐
**Other:**	Yes ☐ No ☐ Not known ☐ **specify:**	Yes ☐ No ☐ Not known ☐ **specify:**	Yes ☐ No ☐ Not known ☐ **specify:**	Yes ☐ No ☐ Not known ☐ **specify:**	Yes ☐ No ☐ Not known ☐ **specify:**

3. IMMUNOSUPPRESSANT MEDICATIONS

	PRE-PREGNANCY	ANTEPARTUM	INTRAPARTUM	POSTPARTUM	AT DISCHARGE
**Mycophenolate**	Yes ☐ No ☐ Not known ☐	Yes ☐ No ☐ Not known ☐	Yes ☐ No ☐ Not known ☐	Yes ☐ No ☐ Not known ☐	Yes ☐ No ☐ Not known ☐
**Azathioprine**	Yes ☐ No ☐ Not known ☐	Yes ☐ No ☐ Not known ☐	Yes ☐ No ☐ Not known ☐	Yes ☐ No ☐ Not known ☐	Yes ☐ No ☐ Not known ☐
**Sirolimus / everolimus**	Yes ☐ No ☐ Not known ☐	Yes ☐ No ☐ Not known ☐	Yes ☐ No ☐ Not known ☐	Yes ☐ No ☐ Not known ☐	Yes ☐ No ☐ Not known ☐
**Tacrolimus/ cyclosporine**	Yes ☐ No ☐ Not known ☐	Yes ☐ No ☐ Not known ☐	Yes ☐ No ☐ Not known ☐	Yes ☐ No ☐ Not known ☐	Yes ☐ No ☐ Not known ☐
**Prednisolone**	Yes ☐ No ☐ Not known ☐	Yes ☐ No ☐ Not known ☐	Yes ☐ No ☐ Not known ☐	Yes ☐ No ☐ Not known ☐	Yes ☐ No ☐ Not known ☐
**Other:**	Yes ☐ No ☐ Not known ☐ **specify:**	Yes ☐ No ☐ Not known ☐ **specify:**	Yes ☐ No ☐ Not known ☐ **specify:**	Yes ☐ No ☐ Not known ☐ **specify:**	Yes ☐ No ☐ Not known ☐ **specify:**
**Other:**	Yes ☐ No ☐ Not known ☐ **specify:**	Yes ☐ No ☐ Not known ☐ **specify:**	Yes ☐ No ☐ Not known ☐ **specify:**	Yes ☐ No ☐ Not known ☐ **specify:**	Yes ☐ No ☐ Not known ☐ **specify:**

4. **Were any other drugs (apart from anti-hypertensive agents and immunosuppressant agents) CEASED for this pregnancy?**

Yes ☐ No ☐ Not known ☐

(If yes)

Choose one

Name of drug ______________________ ☐ Ceased PRIOR to conception OR ☐ Ceased AFTER conception

Name of drug ______________________ ☐ Ceased PRIOR to conception OR ☐ Ceased AFTER conception

5. OTHER TREATMENTS DURING THIS PREGNANCY

5.1. Aspirin Yes ☐ No ☐ Not known ☐

5.2. Erythropoietin Yes ☐ No ☐ Not known ☐
(If yes) Name of agent __________________
Treatment pre-pregnancy? Yes ☐ No ☐ Not known ☐

5.3. Iron therapy Yes ☐ No ☐ Not known ☐
(If yes) Oral Yes ☐ No ☐ Not known ☐
Intravenous Yes ☐ No ☐ Not known ☐

5.4. Blood transfusion Yes ☐ No ☐ Not known ☐
(If yes) Date___/___/______
Number of units __________________

Reason for transfusion
Chronic anaemia Yes ☐ No ☐ Not known ☐
Acute blood loss Yes ☐ No ☐ Not known ☐
Other Yes ☐ No ☐ Not known ☐
(If yes) specify ____

5.5. Heparin (unfractionated or LMW) Yes ☐ No ☐ Not known ☐
(If yes) Name of agent __________________
Indication __________________
Date started ___/___/______

5.6. Corticosteroids (if not previously receiving corticosteroids)
Yes ☐ No ☐ Not known ☐ Not applicable ☐

(If yes) Methylprednisolone Yes ☐ No ☐ Not known ☐
(If yes) Indication __________________
Date started ___/___/______

Oral prednisolone Yes ☐ No ☐ Not known ☐
(If yes) Indication __________________
Date started ___/___/______

Betamethasone/dexamethasone for fetal lung maturity/thrombocytopenia
Yes ☐ No ☐ Not known ☐
(If yes) Indication __________________
Date started ___/___/______

5.7. Intravenous immunoglobulin Yes ☐ No ☐ Not known ☐
(If yes) Indication __________________

5.8. Magnesium sulphate (MgSO4) Yes ☐ No ☐ Not known ☐

SECTION 4

5.9. Antibiotics for TREATMENT of infection (NOT single doses)
Yes ☐ No ☐ Not known ☐
(If yes)
Name of agent ________________
Indication ________________

5.10. Antibiotics for PROPHYLAXIS against infection (NOT single doses)
Yes ☐ No ☐ Not known ☐
If yes
Name of agent ________________
Indication ________________

5.11. Non-steroidal anti-inflammatory drugs
Yes ☐ No ☐ Not known ☐
If yes
Name of agent ________________
Indication ________________

5.12. Folic acid
Yes ☐ No ☐ Not known ☐
If yes
Dose (mg/day) __________

5.13. Did the patient start any other new medications started during this pregnancy (excluding the above, multivitamins, Vitamin D and calcium)?
Yes ☐ No ☐ Not known ☐
If yes
Name of agent/date commenced ____
____

SECTION 5: ANTENATAL, BIRTH AND POSTPARTUM CARE

1. **Did this woman have pre-existing, known hypertension PRIOR to pregnancy?**
Yes ☐ No ☐ Not known ☐
(If yes)

1.1. Hypertension type (*choose one*)
☐ Untreated hypertension: Hypertensive > 140/90 mmHg, not on treatment **OR**
☐ Uncontrolled hypertension: Hypertensive > 140/90 mmHg, on anti-hypertensive therapy **OR**
☐ Controlled hypertension: Normotensive <= 140/90 mmHg, on anti-hypertensive therapy **OR**
☐ Not known

(If no or not known)

1.2. Did this woman develop newly diagnosed hypertension during this pregnancy?
Yes ☐ No ☐ Not known ☐
(If yes) Was hypertension first identified at the booking visit? Yes ☐ No ☐ Not known ☐
Date hypertension first detected: ____/____/_____

2. BLOOD PRESSURE READINGS IN THIS PREGNANCY (HIGHEST)

	PRE-PREGNANCY		AT BOOKING		1ST TRIMESTER (1-13 weeks)		2ND TRIMESTER (14-27 weeks)	
	Value	*Date*	*Value*	*Date*	*Value*	*Date*	*Value*	*Date*
**Systolic (mmHg)**								
**Diastolic (mmHg)**								

	3RD TRIMESTER (28+ weeks)		DELIVERY		DISCHARGE	
	Value	*Date*	*Value*	*Date*	*Value*	*Date*
**Systolic (mmHg)**						
**Diastolic (mmHg)**						

3. ANTENATAL SCREENING AND GROWTH SCANS

Please skip this question and move to question 4 if you have attached deidentified scan data

3.1. **Did this woman have 1st/2nd trimester antenatal screening during this pregnancy?**
Yes ☐ No ☐ Not known ☐
(*If yes)*

	Date	*Gestational age (weeks)*	*Value*
**PAPPA MoM**			
**Nuchal translucency (NT) scan NT mm**			
**β-hCG MoM**			
**Adjusted Down Syndrome Risk**			
**Non-invasive prenatal test (NIPT)**			
**Umbilical artery pulsatility index (UAPI)**			
**Serum placental growth factor (PIGF)**			
**Other abnormalities**			

AMOSS Case ID ________________________

SECTION 5

4. **Did this woman have a fetal anomaly or growth scan during this pregnancy?**
Yes ☐ No ☐ Not known ☐
(If yes) How many scans in total? ______ (number)

Please skip this section and move to question 5 if you have attached deidentified scan data

	1st SCAN		2nd SCAN		3rd SCAN*	
	Value	***%ile***	***Value***	***%ile***	***Value***	***%ile***
**Date of scan**						
**Gestational age (weeks)**						
**Amniotic fluid index (AFI)**	cm		cm		cm	
**Crown-rump length (CRL)**	mm		mm		mm	
**Deepest vertical pocket (DVP)**	cm		cm		cm	
**Estimated fetal weight (EFW)**	grams		grams		grams	
**Biparietal diameter (BPD)**	mm		mm		mm	
**Head circumference (HC)**	mm		mm		mm	
**Abdominal circumference (AC)**	mm		mm		mm	
**Femur Length (FL)**	mm		mm		mm	
**Umbilical artery pulsatility index (UAPI)**						
**Uterine artery resistance index (UARI)**	☐ Normal ☐ Abnormal<br>☐ Unilateral ☐ Bilateral<br>☐ Not known		☐ Normal ☐ Abnormal<br>☐ Unilateral ☐ Bilateral<br>☐ Not known		☐ Normal ☐ Abnormal<br>☐ Unilateral ☐ Bilateral<br>☐ Not known	
**Uterine artery notching**	☐ Mild ☐ Severe<br>☐ Unilateral ☐ Bilateral<br>☐ Not known		☐ Mild ☐ Severe<br>☐ Unilateral ☐ Bilateral<br>☐ Not known		☐ Mild ☐ Severe<br>☐ Unilateral ☐ Bilateral<br>☐ Not known	

**If more than three scans please attach deidentified results at the end of the survey.*

5. **Was pre-eclampsia diagnosed in this pregnancy?**
Yes ☐ No ☐ Not known ☐
(If yes) Date of onset of pre-eclampsia ____/____/_____

Pre-eclampsia features (present at any time)

5.1. Hypertension (≥140/90 mmHg) Yes ☐ No ☐ Not known ☐
5.2. Proteinuria (urine P:Cr > 30 mg/mmol) Yes ☐ No ☐ Not known ☐
5.3. Low platelets (< 100,000 x 109/L) Yes ☐ No ☐ Not known ☐
5.4. High uric acid Yes ☐ No ☐ Not known ☐
(greater than the upper limit for local lab reference range for gestational age)
5.5. Renal impairment Yes ☐ No ☐ Not known ☐
5.6. Liver function derangement Yes ☐ No ☐ Not known ☐
5.7. Headache Yes ☐ No ☐ Not known ☐
5.8. Upper abdominal or Right Upper quadrant pain Yes ☐ No ☐ Not known ☐
5.9. Vomiting Yes ☐ No ☐ Not known ☐
5.10. Neurological symptom (without seizures) Yes ☐ No ☐ Not known ☐
5.11. Seizures (Eclampsia) Yes ☐ No ☐ Not known ☐
5.12. Fetal growth restriction (<10th centile) Yes ☐ No ☐ Not known ☐
5.13. Oligohydramnios Yes ☐ No ☐ Not known ☐
5.14. Abnormal umbilical artery Dopplers Yes ☐ No ☐ Not known ☐

SECTION 5

6. **Did this woman develop new-onset proteinuria in this pregnancy (if not detected prior to pregnancy or at booking)**?
Yes ☐ No ☐ Not known ☐
(*If yes)* Date proteinuria was first detected: ____/____/_____

7. **Did this woman have a urinary tract infection during this pregnancy?**
Yes ☐ No ☐ Not known ☐
(If yes) Date ___/___/______ or gestational age
Organism _________________
Treatment _________________

8. **Did this woman have asymptomatic bacteriuria during this pregnancy?**
Yes ☐ No ☐ Not known ☐
(*If yes)* Date ___/___/______
Treatment _________________

9. **Was this woman admitted early (pre-37 weeks) for delivery?**
Yes ☐ No ☐
(*If yes)* Gestation admitted ___________ weeks OR Date admitted ___/___/_____

10. **Monitoring During Delivery (beyond basic vital sign monitoring)**

**10.1.**	BP monitoring	Yes ☐ No ☐ Not known ☐
**10.2.**	Urine output	Yes ☐ No ☐ Not known ☐
**10.3.**	Arterial line	Yes ☐ No ☐ Not known ☐
**10.4.**	CVP line	Yes ☐ No ☐ Not known ☐
**10.5.**	Cardiac monitoring	Yes ☐ No ☐ Not known ☐
**10.6.**	CTG	Yes ☐ No ☐ Not known ☐
**10.7.**	Other	Yes ☐ No ☐ Not known ☐

(If yes) specify _________________________________

11. **Did this woman receive an indwelling bladder catheter during labour/postpartum?**
Yes ☐ No ☐ Not known ☐

12. **Did any of the following events/interventions occur during labour or delivery?**

12.1. Oliguria (urine output <30ml/hour for > 2 hours) Yes ☐ No ☐ Not known ☐

12.2. Severe hypertension (either BP ≥160 systolic or ≥110 diastolic (mmHg)) Yes ☐ No ☐ Not known ☐

12.3. High potassium (≥6.0 mmol/L or requiring treatment) Yes ☐ No ☐ Not known ☐

12.4. Low blood pressure <90 mmHg Yes ☐ No ☐ Not known ☐
(If yes) Fluid resuscitation only Yes ☐ No ☐ Not known ☐
Inotropes Yes ☐ No ☐ Not known ☐

12.5. Blood/platelet transfusion Yes ☐ No ☐ Not known ☐
(If yes) Number of units ____________________ (RBC/platelets)

13. Did any of the following events/interventions occur post-partum prior to discharge?

13.1. Oliguria (urine output <30ml/hour for > 2 hours) Yes ☐ No ☐ Not known ☐

13.2. Severe hypertension (either BP ≥160 systolic or ≥110 diastolic (mmHg)) Yes ☐ No ☐ Not known ☐

13.3. High potassium (≥6.0 mmol/L or requiring treatment) Yes ☐ No ☐ Not known ☐

13.4. Low blood pressure <90 mmHg Yes ☐ No ☐ Not known ☐
(If yes) Fluid resuscitation only Yes ☐ No ☐ Not known ☐
Inotropes Yes ☐ No ☐ Not known ☐

13.5. Blood/platelet transfusion Yes ☐ No ☐ Not known ☐
(If yes) Number of units ____________________ (RBC/platelets)

14. Did this woman receive non-steroidal anti-inflammatory medication postpartum prior to discharge?
Yes ☐ No ☐ Not known ☐
(*If yes*) Name of agent _________________ Indication ______________________

15. Was there a plan for follow up with the following medical providers?

15.1. GP Yes ☐ No ☐ Not known ☐
15.2. High-risk clinic Yes ☐ No ☐ Not known ☐
15.3. Nephrologist Yes ☐ No ☐ Not known ☐
15.4. Other Yes ☐ No ☐ Not known ☐

16. Is there any other relevant information regarding this patient? Please provide details.

End of Survey. Thank you!
