# Supplementary material for: Serious kidney disease in pregnancy: an Australian national cohort study protocol
Source: BMC Nephrol. 2019 Jun 25;20:230. doi: 10.1186/s12882-019-1393-z (PMC6593486; doi:10.1186/s12882-019-1393-z)
Supplement: Supplementary file 3 — Main outcome measurements: A list of the main outcome measures that will form the basis of data analysis. (DOCX 12 kb) [file 12882_2019_1393_MOESM3_ESM.docx]

**Additional file 3 Main outcome measurements**

|  | **Outcome measurements** |
| --- | --- |
| Mortalities | Maternal mortality  Stillbirth  Neonatal death |
| Morbidities –  Obstetrical/ Maternal outcomes | Thromboembolic events  Amniotic embolism  Antepartum haemorrhage  Postpartum haemorrhage  Preterm premature rupture of membranes  Induction of labour  Caesarean section  Preterm labour (spontaneous and induced)  Preeclampsia  Placental previa  Placental abruption  Hysterectomy  Sepsis  Long hospital stay |
| Morbidities –  Renal | Deterioration of renal function during pregnancy  Graft dysfunction  Commencement of dialysis  Timing of renal function deterioration during pregnancy |
| Morbidities –  infant outcomes | Intrauterine growth restriction  Congenital malformation  Low birthweight  Small for gestational age  Preterm birth  Resuscitation  Apgar score  Admission to a neonatal intensive care unit  Neonatal hospital stay |
