# Supplementary material for: Serious kidney disease in pregnancy: an Australian national cohort study protocol
Source: BMC Nephrol. 2019 Jun 25;20:230. doi: 10.1186/s12882-019-1393-z (PMC6593486; doi:10.1186/s12882-019-1393-z)
Supplement: Supplementary file 4 — Selected covariates: The list of variables that we expect to be associated with the outcomes. (DOCX 13 kb) [file 12882_2019_1393_MOESM4_ESM.docx]

**Additional file 4: selected covariates:**

|  | **Covariates** |
| --- | --- |
| Obstetrical/ antenatal/demographic | Type of initial model of care  Change in the model pf care  Remoteness  Indigenous status  Country of birth  Public or private care  Smoking status  BMI  Pre-existing medical conditions  Pre-existing obstetric conditions  Parity  Plurality  Current medical conditions  Current obstetric complications  Medications used during pregnancy |
| Renal | Underlying cause of kidney disease  Baseline renal function  Number of kidney transplants  Transplant source  Dialysis modality during pregnancy  Number of dialysis sessions per week  Number of hours per dialysis session  Site of dialysis (hospital, home, satellite Unit) |
